# Quantification of Uncertainties on the Critical Buckling Load of Columns under Axial Compression with Uncertain Random Materials

**DOI:** 10.3390/ma12111828

**Published:** 2019-06-05

**Authors:** Hai-Bang Ly, Christophe Desceliers, Lu Minh Le, Tien-Thinh Le, Binh Thai Pham, Long Nguyen-Ngoc, Van Thuan Doan, Minh Le

**Affiliations:** 1 University of Transport Technology, Hanoi 100000, Vietnam; binhpt@utt.edu.vn; 2 Laboratoire Modélisation et Simulation Multi Echelle, Université Paris-Est, MSME UMR 8208, 77420 Champs-sur-Marne, France; christophe.desceliers@u-pem.fr; 3 Faculty of Engineering, Vietnam National University of Agriculture, Gia Lam, Hanoi 100000, Vietnam; 4 Institute of Research and Development, Duy Tan University, Da Nang 550000, Vietnam; 5 University of Transport and Communications, Hanoi 100000, Vietnam; nguyenngoclong@utc.edu.vn; 6 NTT Hi-Tech Institute, Nguyen Tat Thanh University, Ho Chi Minh City 700000, Vietnam; dvthuan@ntt.edu.vn (V.T.D.); vuongminhle09@gmail.com (M.L.)

**Keywords:** uncertainty quantification, critical buckling load, probabilistic model, finite element method, random elasticity matrix, Newton-Raphson

## Abstract

This study is devoted to the modeling and simulation of uncertainties in the constitutive elastic properties of material constituting a circular column under axial compression. To this aim, a probabilistic model dedicated to the construction of positive-definite random elasticity matrices was first used, involving two stochastic parameters: the mean value and a dispersion parameter. In order to compute the nonlinear effects between load and lateral deflection for the buckling problem of the column, a finite element framework combining a Newton-Raphson solver was developed. The finite element tool was validated by comparing the as-obtained critical buckling loads with those from Euler’s formula at zero-fluctuation of the elasticity matrix. Three levels of fluctuations of material uncertainties were then propagated through the validated finite element tool using the probabilistic method as a stochastic solver. Results showed that uncertain material properties considerably influenced the buckling behavior of columns under axial loading. The coefficient of variation of a critical buckling load over 500 realizations were 15.477%, 26.713% and 41.555% when applying dispersion parameters of 0.3, 0.5 and 0.7, respectively. The 95% confidence intervals of column buckling response were finally given. The methodology of modeling presented in this paper is a potential candidate for accounting material uncertainties with some instabilities of structural elements under compression.

## 1. Introduction

Various parameters of structural elements are uncertain in nature, for instance, the length of structural components [1]; geometry of the cross-section [2]; boundary conditions [3]; loads [4]; and especially mechanical properties of materials [5,6,7,8,9,10,11,12]. Indeed, uncertainty in Young’s modulus, Poisson’s ratio and yield strength of materials constituting structural elements has been experimentally explored in the literature, such as by Shi et al. [5] for hollow circular steel tubes, Ma et al. [1] for I-section steel columns, Cao et al. [7] for welded T-section structural elements, Jamaluddin et al. [13] for elliptical concrete filled columns and Vu et al. [14] for circular concrete filled steel tubes. The uncertainties of materials’ properties exhibited an important effect on the force-resistance of structural elements regarding the initial design [15], particularly for structural members under axial compression. As an example, Shi et al. [5] found that the ultimate stress of steel circular tubes under axial load was smaller, that is, 0.8–0.9 times, than the corresponding stress calculated by the two standards Eurocode BS EN 1993-1-12 [16] and ANSI/AISC 360-10 [17] for steel structures. This means instability of steel tubes appears even before estimation that is provided by the two standards mentioned. In order to fully characterize the mechanical behavior of structural elements, it is necessary to take into account the uncertainties of material properties in modeling and simulation, as they become relevant.

In the last three decades, uncertainty quantification has received a tremendous amount of attention from researchers around the world in the field of computational mechanics, especially in mechanical and structural engineering [18,19]. A stochastic model based on Karhunen-Loeve expansion was early proposed by Spanos et al. [20] for modeling the random rigidity of a cantilever beam. In another attempt, Soize [21,22] developed a theory of random matrices ensembles based on the theory of information with the maximum entropy principle. The author was the first to obtain nonparametric probabilistic models for the generalized matrices of mass, dumping and stiffness in structural dynamics. Besides, the reliability of structural components in the presence of random mechanical properties, subjected to random loads, was investigated by Der Kiureghian et al. [23], using finite element method. Within the context of multiscale modeling, Vu-Bac et al. [24] studied the uncertainty propagation induced by input parameters on the effective properties of nano-composite polymer based on molecular dynamics simulations. Moreover, Akmar et al. [25] investigated the sensitivity analysis of uncertain input parameters that affects the effective mechanical behavior of dry textiles under different deformations. The mechanical properties of masonry structures have been stochastically represented by Falsone et al. [10] based on image processing techniques. A hyper-elastic constitutive model of laminated composite has been identified by Staber et al. [26] using non-Gaussian random fields. Regarding uncertainty modeling in structural analysis, Castaldo et al. [27,28] proposed a probabilistic quantification for safety factors of reinforced concrete members based on the use of the nonlinear finite element technique. Manifold studies of Haukaas et al. [29], Most [30] and Ben Ftima et al. [31] also investigated model uncertainty in structural engineering using a Bayesian approach for assessment of model parameters. Other sources of uncertainties in structural analysis were discussed in the work of Der Kiureghian et al. [32]. So far, studies involving uncertainty quantification have been able to explain and predict the reliability of mechanical responses of structural elements.

Nevertheless, in regard to buckling analysis of columns under axial compression, most of the models previously developed are, until now, deterministic and have presumed that material uncertainty has no effect on the critical buckling load. Several studies involving uncertainty quantification have been recently introduced in the literature. Korkmaz et al. [33] proposed a model based on fuzzy logic for accounting material uncertainty of reinforced concrete columns under axial loads. Buckling uncertainty of steel pipelines in contact with elastic soil was investigated by Athmani et al. [34]. In the work of Gao et al. [35], the authors introduced a Chebyshev surrogate model to characterize the dynamic buckling of beam-column structures. Last but not least, buckling uncertainty of carbon nanotube-reinforced composite under compressive stress was reported in the work of Pouresmaeeli et al. [36], using Galerkin’s method.

Despite all of these efforts, it is not always possible to investigate the highly nonlinear relationship between load–lateral deflection of columns under axial loading, as reported in the literature [1,2,5,6,11,37,38,39]. Such nonlinear phenomenon (large displacement when the load increment is small) made the instability analysis of structural components under compressive stress more complicated [40,41]. Yang et al. [4] used the commercial finite element software Abaqus to investigate the buckling problem for I-section steel columns under axial loading. Jiang et al. [39] applied Abaqus in order to simulate the buckling behavior of pseudo-elastic Nicken Titanium alloy tubes. Shi et al. [5] employed another commercial software, namely Ansys, to generate 60 finite element buckling configurations based on 24 initial experimental tests. However, commercial software programs are not compatible with large datasets when propagating material uncertainty on buckling responses [42,43]. Within the context of uncertainty propagation through partial differential equations, the development of code in programming language is indispensable.

The main purpose of this study is to quantify the impact of uncertainties in material properties on the critical buckling load of columns under axial compression. With this aim, uncertainty in material properties was modeled using the ensemble of random matrices, constructed by Soize [21,22,44]. A nonlinear finite element tool involving the Newton-Raphson solver was implemented and validated for tracking the mechanical response of columns. Monte Carlo method was finally carried out as a stochastic solver with such in-house numerical tools for statistically estimating column buckling capacity in the presence of material uncertainties.

## 2. Materials and Methods

### 2.1. Description of the Considered Column and Its Material Properties

**Figure 1:**
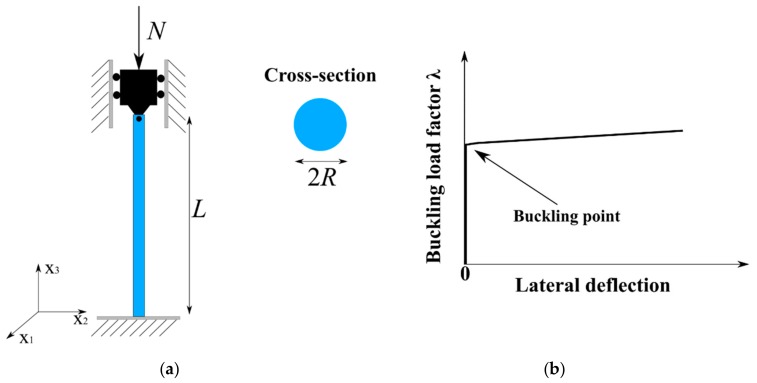
Description of column under axial loading: (**a**) schematization of the column considered in this study, including length, geometry of cross-section, fixed-pinned boundary conditions and axial loading at the top surface, and (**b**) typical mechanical response of columns under axial compression (buckling load factor versus lateral deflection).

In this study, a fixed-pinned circular column subjected to axial load is considered. Geometry of the column is schematized in Figure 1a, including the length *L* (in m), the radius *R* (in m) of the circular cross-section. The column was subjected to axial force *N* (in kN). The load factor involving critical Euler’s buckling load was defined by the following equation [45]:

(1) $$\lambda =\frac{N}{N^{\mathrm{Euler}}},$$

where *N*^Euler^ was defined such as [40,46]:

(2) $$N^{\mathrm{Euler}}=\frac{\pi^{2}EI}{{\left(KL\right)}^{2}},$$

where *E* is the Young’s modulus of material (in GPa), *I* is the minimum area moment of inertia of the cross section of the column (in m^4^), *L* is the length of the column (in m) and *K* is the column effective length factor. In this case of circular cross-section and fixed-pinned end boundary conditions, $I=\pi R^{4}/4$ and *K* = 0.7. Slenderness of the circular column was introduced such as:

(3) $$SLD=\frac{2L}{R}$$

Lateral deflection of the column Δr (in %) was defined using the following equation:

(4) $$\Delta r=\frac{r}{L}\times 100,$$

where *r* is the maximum lateral deflection in m over the centerline of the column. For such a column under axial compression, typical buckling behavior was reported by many researchers in the literature [2,5,6,8]. A schematization of the nonlinear relationship between load and lateral deflection of the column is presented in Figure 1b. The elastic medium of the column was assumed to be homogeneous, for which the typical symmetric positive-definite elasticity matrix was modeled by a random matrix [C] with values into the set of (6 × 6) real-valued positive definite matrices, which can be written as in Equation (5). In Equation (5), elements ${\left[C\right]}_{\mathrm{ij}}$ are a set of statistically dependent real-valued random variables for which the probabilistic models should be constructed.

(5) $$\left[C\right]=\left[\begin{array}{cccccc} C_{11} & C_{12} & C_{13} & C_{14} & C_{15} & C_{16}\\ & C_{22} & C_{23} & C_{24} & C_{25} & C_{26}\\ & & C_{33} & C_{34} & C_{35} & C_{36}\\ & & & C_{44} & C_{45} & C_{46}\\ & \mathrm{Sym}. & & & C_{55} & C_{56}\\ & & & & & C_{66} \end{array}\right]$$

In this paper, it is assumed that, on average, the random elastic medium is isotropic and is represented by the (6 × 6) symmetric positive-definite matrix $\left[{\underline{C}}^{\mathrm{isotropic}}\right]$, defined as

(6) $$\left[{\underline{C}}^{\mathrm{isotropic}}\right]=\left[\begin{array}{cccccc} k+4\mu /3 & k-2\mu /3 & k-2\mu /3 & 0 & 0 & 0\\ & k+4\mu /3 & k-2\mu /3 & 0 & 0 & 0\\ & & k+4\mu /3 & 0 & 0 & 0\\ & & & 2\mu & 0 & 0\\ & \mathrm{Sym}. & & & 2\mu & 0\\ & & & & & 2\mu \end{array}\right]$$

where

(7) $$k=\frac{E}{3(1-2\nu )},\mu =\frac{E}{2(1+\nu )}$$

which involves the mean bulk modulus *k* and the mean shear modulus *µ* that are defined with respect to the mean Young modulus *E* = 210 GPa and the mean Poisson’s ratio = 0.28 by Equation (7).

### 2.2. Probabilistic Model for Random Matrix

In this paper, a probabilistic model of the random elasticity matrix [C] was constructed in using the ensemble of random matrices SE^+^ introduced by Soize [21,22]. The construction of the ensemble SE^+^ is based on the information theory and on the use of the Maximum Entropy Principe. The probability distribution of the random matrices were derived analytically in solving an optimization problem under statistical constraints that correspond to the available information concerning the random matrices. Such an available statistical information is the following,

- Random matrix [C] in ensemble SE^+^ is positive-definite almost surely with values in $M_{6}^{+}(\mathbb{R})$;
- Mean value of [C] is the 6 × 6 given and equal to $\left[{\underline{C}}^{\mathrm{isotropic}}\right]$:
  (8) $$\left[\underline{C}\right]=E\left\{\left[C\right]\right\}=\left[{\underline{C}}^{\mathrm{isotropic}}\right],$$
  where $E\left\{\right\}$ denotes the mathematical expectation operator.
- Inverse of random matrix [C] is almost-surely a second-order random variable:
  (9) $$E\left\{\ln\left(\det\left(\left[C\right]\right)\right)\right\}<+\infty$$

The probability density function of the random matrix [C] is then derived as well as an *adhoc* algebraic representation for the random matrix [C]. We have, almost certainly:

(10) $$\left[C\right]={\left[{\underline{L}}_{C}\right]}^{T}\left[G\right]\left[{\underline{L}}_{C}\right]$$

where ${[\underline{L}}_{C}]$ is a (6 × 6) upper triangular matrix constructed from the Cholesky factorization of the deterministic matrix $\left[{\underline{C}}^{\mathrm{isotropic}}\right]={\left[{\underline{L}}_{C}\right]}^{T}{\underline{L}}_{C}$ in which upper script T means the transpose operator of the matrix. Random (6 × 6) positive-definite valued random matrix [G] added into Equation (10) can be written, in using its Cholesky factorization, almost certainly as:

(11) $${[G]=[L}_{G}{]}^{T}{[L}_{G}]$$

In which ${[L}_{G}]$ is a (6 × 6) upper triangular matrix-valued random variable. It has been proved that [C] belongs to ensemble SE^+^ if and only if the elements ${{[L}_{G}]}_{\mathrm{ij}}$ of random matrix ${[L}_{G}]$ are real-valued random variables, such that:

(12) $${\left[L_{G}\right]}_{\mathrm{ij}}={\sigma U}_{\mathrm{ij}}\;\mathrm{for}\;i<j\leq 6$$

(13) $${\left[L_{G}\right]}_{\mathrm{ii}}=\sigma \sqrt{2V_{j}}\;\mathrm{for}\;i<j\leq 6$$

in which $\sigma =\delta_{G}7^{-1/2}$ in which δ*_G_* is a given dispersion coefficient, where $U_{\mathrm{ij}}$ and $V_{j}$ with 1≤i<j≤6 are a set of statistically independent real-valued random variables with normal probability distribution for $U_{\mathrm{ij}}$ (normalized and centered) and with gamma probability distribution with coefficient: $\alpha_{j}=\frac{7}{2}\delta^{2}+\frac{\left(1-j\right)}{2}$ for $V_{j}$.

First, we note that for such a probabilistic model, entries $C_{\mathrm{ij}}$ of the random matrix $\left[C\right]$ are statistically dependent real-valued random variables whose probabilistic model is not Gaussian. This model is different to the probabilistic model obtained by taking [C] into the Gaussian orthogonal ensemble (GOE). Second, the algebraic representation of [C] given by Equations (10)–(13) allows the use of Monte Carlo numerical method as a very straightforward stochastic solver. Finally, it is quite remarkable to note that this probabilistic model, based on the theory of information, depends only on a minimal set of parameters, the dispersion coefficient δ*_G_* and the entries of the mean elasticity matrix $\left[{\underline{C}}^{\mathrm{isotropic}}\right]$. Finally, for δ*_G_* = 0, the probability distribution of [C] is a Dirac distribution, and consequently [C] is almost certainly equal to its mean value $\left[{\underline{C}}^{\mathrm{isotropic}}\right]$. In addition, it has also been proved that the dispersion coefficient δ*_G_* is bounded by $\sqrt{7/11}$.

### 2.3. Finite Element Formulation

The equations of the boundary value problems in finite displacements are written

(14) $$\begin{array}{l} \forall x_{0}\in \Omega_{0},divx_{0}(F_{0}(x_{0})\Pi_{0}(x_{0}))=0\\ \forall x_{0}\in \Sigma_{0},F_{0}(x_{0})\Pi_{0}(x_{0})n_{0}(x_{0})=\lambda f_{s}\\ \forall x_{0}\in \Gamma_{0},u_{0}(x_{0})=0 \end{array}$$

where $F_{0}$ is the gradient of deformation; $\Pi_{0}$ is the second Piola Kirchhoff stress tensor; $u_{0}$ is the displacement field of the natural configuration which occupies the domain $\Omega_{0}$; $\lambda f_{s}$ is the force applied on the surface $\Sigma_{0}$ of the boundary of $\Omega_{0}$ where λ is a load factor and $f_{s}$ is a reference force; $n_{0}$ is the exterior normal vector of $\Omega_{0}$ on the edge $\partial \Omega_{0}$ of $\Omega_{0}$. The medium that occupies the domain $\Omega_{0}$ is assumed to be linear elastic in finite displacement. It is then assumed that the constitutive equations finite displacement yields linear equations between the stress tensor of Piola Kirchhoff $\Pi_{0}$ and the strain Green Lagrange $E_{0}$ is written as

(15) $$\Pi_{0}=A_{0}:E_{0}$$

in which $A_{0}(x_{0})$ is the fourth order elastic tensor which satisfies the following properties:

(16) $$\begin{array}{l} {\left\{A_{0}(x_{0})\right\}}_{ijkh}={\left\{A_{0}(x_{0})\right\}}_{jikh}={\left\{A_{0}(x_{0})\right\}}_{ijhk}={\left\{A_{0}(x_{0})\right\}}_{khij}\\ {\left\{A_{0}(x_{0})\right\}}_{ijkh}{\left\{X_{0}\right\}}_{ij}{\left\{X_{0}\right\}}_{kh}\geq c{\left\{X_{0}\right\}}_{ij}{\left\{X_{0}\right\}}_{kh} \end{array}$$

for any real symmetric second order tensor X and for a given real c> 0. For finite element analysis, let us introduce $C_{ad}$ the set of functions u defined on $\Omega_{0}$ with the values in ${\mathbb{R}}^{3}$ and sufficiently regular on $\Omega_{0}$. Let $C_{0}$ the subset of functions u of $C_{ad}$ whose trace is null on $\Gamma_{0}$. The weak formulation of the boundary value problems can be written as:

Find $u\in C_{0}$ such that for all $v\in C_{0}$

(17) $$k_{e}(u,v)+k_{2,1}(u,u,v)+k_{2,2}(v,u,u)+k_{3}(u,u,u,v)=\lambda l(v)$$

In Equation (17), the symmetric bilinear operator $k_{e}$ is defined as

(18) $$k_{e}(u,v)=\int_{\Omega_{0}}\left\{A_{0}:\frac{\partial u}{\partial x_{0}}\right\}:\frac{\partial v}{\partial x_{0}}dx_{0}$$

This is a symmetric definite-positive bilinear form on $C_{0}\times C_{0}$. Furthermore, in Equation (19), the three other multi-linear forms $k_{2,1}$ and $k_{2,2}$ (defined on $C_{0}\times C_{0}\times C_{0}$) and $k_{3}$ (defined on $C_{0}\times C_{0}\times C_{0}\times C_{0}$) are defined as

(19) $$\begin{array}{l} k_{2,1}(u,v,w)=\frac{1}{2}\int_{\Omega_{0}}\left\{A_{0}:\left(\frac{\partial u^{T}}{\partial x_{0}}\frac{\partial v}{\partial x_{0}}\right)\right\}:\frac{\partial w}{\partial x_{0}}dx_{0}\\ k_{2,2}(u,v,w)=\int_{\Omega_{0}}\left\{A_{0}:\frac{\partial u}{\partial x_{0}}\right\}:\left(\frac{\partial v^{T}}{\partial x_{0}}\frac{\partial w}{\partial x_{0}}\right)dx_{0}\\ k_{3}(u,v,w,r)=\frac{1}{2}\int_{\Omega_{0}}\left\{A_{0}:\left(\frac{\partial u^{T}}{\partial x_{0}}\frac{\partial v}{\partial x_{0}}\right)\right\}:\left(\frac{\partial w^{T}}{\partial x_{0}}\frac{\partial r}{\partial x_{0}}\right)dx_{0} \end{array}$$

Finally, the linear form l(v) defined on $C_{0}$ is defined as

(20) $$l(v)=\int_{x\in \Sigma_{0}}v(x_{0})\cdot f_{s}(x_{0})dS(x)$$

where the dot means the Euclidian inner product. Hereafter, the existence and uniqueness of a solution to the problem is assumed.

### 2.4. Newton-Raphson Technique

The weak forms of the problem can be written as operators for the sake of simplicity when discussing the numerical scheme for constructing a solution to the problem. Let us introduce the set $C_{0}^{\prime }$ of the linear forms defined on $C_{0}$ as well as the operator $K_{e}$ de*fi*ned on $C_{0}$ with values in $C_{0}^{\prime }$ such that:

(21) $$\langle K_{e}(u),v\rangle =k_{e}(u,v)$$

where $\langle ,\rangle$ is the duality bracket between $C_{0}^{\prime }$ and $C_{0}$. Let us also introduce three multi-linear operators $K_{2,1}$, $K_{2,2}$ (defined on $C_{0}\times C_{0}\times C_{0}$) and $K_{3}$ (defined on $C_{0}\times C_{0}\times C_{0}\times C_{0}$) with values in $C_{0}^{\prime }$ such that

(22) $$\begin{array}{l} \langle K_{2,1}(u,v),w\rangle =k_{2,1}(u,u,v)\\ \langle K_{2,2}(v,u),w\rangle =k_{2,2}(v,u,u)\\ \langle K_{3}(u,v,w),r\rangle =k_{3}(u,u,u,v) \end{array}$$

Let $q_{ext}$ be also the linear form in $C_{0}^{\prime }$ defined as

(23) $$\langle q_{ext},v\rangle =l(v)$$

Written in term of operators, Equation (17) is rewritten as

(24) $$K_{e}(u)+K_{2,1}(u,u)+K_{2,2}(u,u)+K_{3}(u,u,u)=\lambda q_{ext}$$

which can formally be rewritten as

(25) $$g(u,\lambda )=r_{int}(u)-\lambda q_{ext}=0$$

where

(26) $$r_{int}=K_{e}(u)+K_{2,1}(u,u)+K_{2,2}(u,u)+K_{3}(u,u,u)$$

Let us now consider a physical system, described in the previous section, whose deformed configuration is represented by the displacement field u. The physical system is submitted to external loads which are proportional to load factor λ. It is assumed that u and load factor λ are the solutions of a set non-linear equations that can be written as:

(27) g(u,λ)=0

Let $K_{T}(u,\lambda )$ be the tangent operator of u↦g(u,λ) such that, for all v,

(28) $$K_{T}(u,\lambda )v=\underset{h\to 0}{\lim}\frac{1}{h}(g(u+hv,\lambda )-g(u,\lambda ))=\underset{h\to 0}{\lim}\frac{1}{h}(r_{int}(u+hv)-r_{int}(u))$$

Consequently, $K_{T}(u,\lambda )$ is independent of the load factor λ, and thus it can be rewritten as $K_{T}(u)$. The Newton-Raphson method is an effective method that allows an accurate approximation of solution u to be computed for given values of λ. The principle of the method is to compute the increment Δu, given an increment Δλ of load factor such that:

(29) g(u+Δu,λ+Δλ)=0

where (u,λ) is a known solution of the above equation which has been previously computed. An iterative scheme is used to compute Δu as the limit of the sequence ${\left\{\Delta_{k}u\right\}}_{k}\geq 0$ in which $\Delta_{k}u$ is defined by the following recurrence equation:

(30) $$\Delta_{k}u=\Delta_{k-1}u+\delta_{k}u$$

where

(31) $$\begin{array}{l} \Delta_{k}u=K_{T}(u_{k-1}{)}^{-1}[(\lambda +\Delta \lambda )q_{ext}-r_{int}(u_{k-1})]\\ u_{k-1}=u+\Delta_{k-1}u \end{array}$$

### 2.5. Monte Carlo Method

Monte Carlo method has been largely used as a very powerful stochastic solver in various domains of science, especially for uncertainty quantification [47,48]. Stochastic analysis involving Monte Carlo simulations as a stochastic solver has been reported in many works, for example, for structural dynamics [49,50,51], in vascular mechanics [52,53,54], for composite materials [15,55,56,57,58], for model reduction [59,60], for concrete structures [61,62], for hyper-elastic materials [26,63], and for heat transfer problems [64]. Such techniques, based on statistically independent sampling, are extremely efficient and powerful for calculating the statistical quantities that measure the propagation of the uncertainty of input parameters on the output results. The method could provide good statistical investigation and also has a strong capability in parallelization computing [47,65,66,67], especially in combination with a finite element model [19]. Convergence analysis of the Monte Carlo method with respect to the number of statistically independent samples is carried out by studying the convergence of the following function [57,68,69]:

(32) $$N_{\mathrm{MC}}\mapsto f_{\mathrm{conv}}\left(N_{\mathrm{MC}}\right)=\frac{1}{\underline{W}}\frac{1}{N_{\mathrm{MC}}}\sum_{i=1}^{N_{\mathrm{MC}}}W_{i},$$

where $\underline{W}$ is the mean value of a given random variable W and $N_{\mathrm{MC}}$ is the number of statistically independent samples $W_{1},\ldots ,W_{N_{MC}}$ of random variable W. Such a convergence estimator also provides efficient information about the time-consuming process within a context of reliable statistical analysis of the results.

## 3. Methodology for Modeling

**Figure 2:**
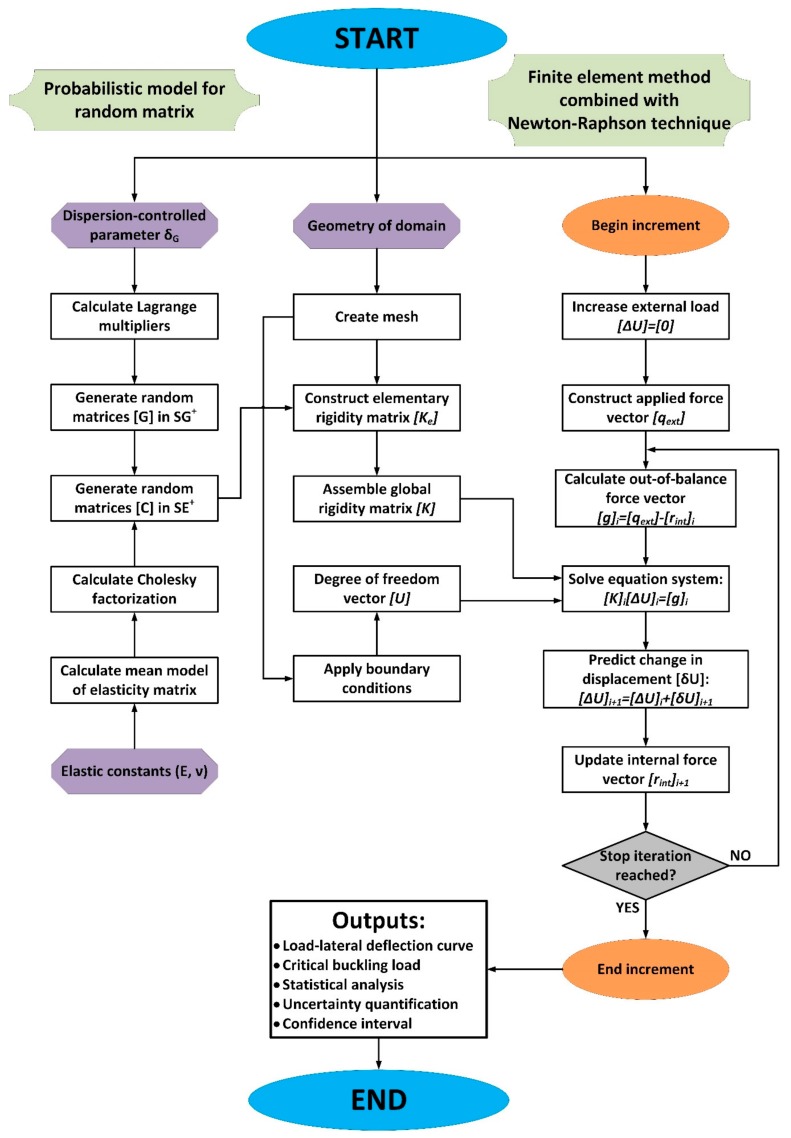
Methodology modeling of the present study including probabilistic model for random matrix and finite element tool combined with the Newton-Raphson technique.

In this study, the methodology for modeling and simulation (Figure 2) was constructed, involving: (i) a probabilistic model for generating uncertainties in material elasticity matrix; and (ii) a finite element model for tracking equilibrium of column under axial compression using the Newton-Raphson method. Dispersion coefficient δ_G_ and elastic constants of materials such as Young’s modulus and Poisson’s ratio are the inputs of the probabilistic model. The construction of this probabilistic model is presented in Section 2.2. Regarding the finite element tool, the code is developed in Matlab for solving the boundary value problem of partial differential equations defined in Section 2.3. The Newton-Raphson method was implemented in order to solve nonlinear equations for the column under compressive stress. The Monte Carlo method was also carried out as a stochastic solver for quantifying the propagation of uncertainties related to the random elasticity matrix on the buckling response of the columns. Finally, statistical analysis was performed to quantify significant information, including the probability density function of the critical buckling load and the 95% confidence intervals.

## 4. Results and Discussion

In this study, the column considered was discretized using linear 4-node tetrahedron—1 integration point elements (Figure 4a). The convergence of the finite element mesh with respect to the number of elements was analyzed by increasing the number of nodes in the domain $\Omega_{0}$. In this work, 12 elements along the radial direction and 220 elements along the length of column were adopted as optimum. Finally, the column was discretized by a total of 44,154 elements (corresponding to 29,568 degrees of freedom). In order to simulate fixed-pinned end boundary conditions of the columns, all nodes at the bottom end of the mesh were fixed, while only axial translation of the center point at the top end was permitted. In the finite element code, lateral translations in both the *x*-axis and *y*-axis of the center point at the top surface were set to be zero, while all other nodes at the top surface were set to be free. Uniformly distributed load was applied at the top end of the column. Since triangular mesh was not regular at the top surface (Figure 4d), that is, the nodal applied force was not exactly the same from one node to another. That way, the instability of column was triggered.

### 4.1. Validation of Numerical Tool

**Figure 3:**
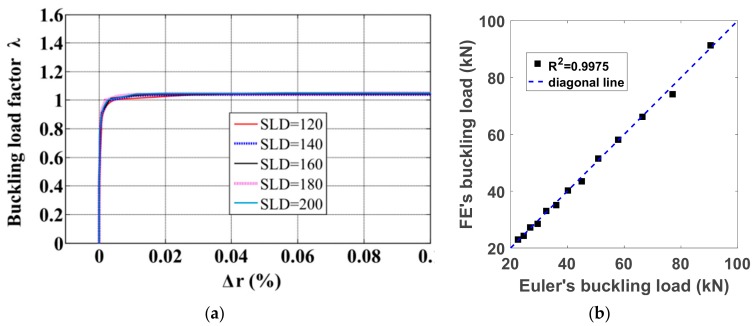
Buckling response of columns under axial compression: (**a**) load factor versus lateral deflection and (**b**) comparison between critical buckling loads obtained from finite element tool and Euler’s formula, for columns with *SLD* ranging from 120 to 200, respectively.

**Figure 4:**
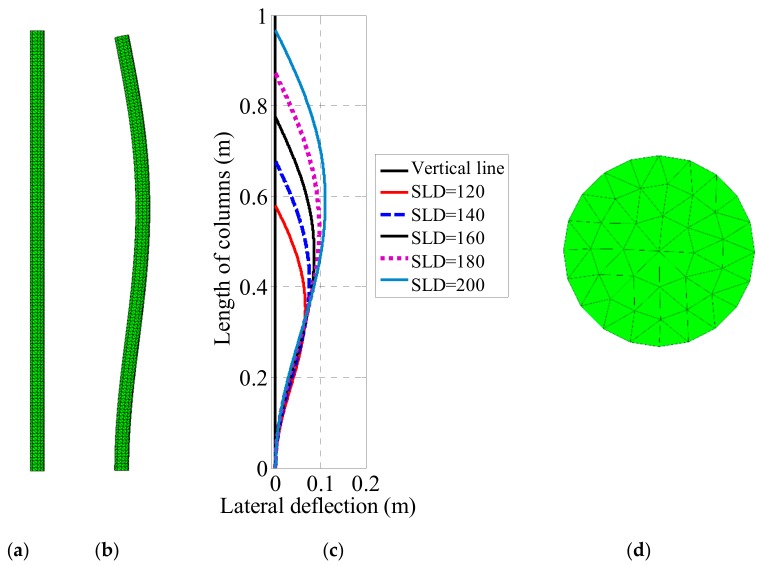
(**a**) Initial and (**b**) deformed tetrahedron meshes of 120 *SLD* column under axial loading, (**c**) deformed centerline of columns with *SLD* = 120, 140, 160, 180 and 200, respectively, and (**d**) visualization of the triangular mesh at the top surface for the case SLD = 120.

In this section, 13 fixed-pinned columns of slenderness ranging from 120 to 240 were investigated using the in-house finite element tool in order to compare the results with those obtained analytically using Euler’s equation [40,45]. Details of the geometry of the 13 columns are given in Table 1. The Young’s modulus of all columns was 210 GPa while the Poisson’s ratio was 0.28 (i.e., associated to the mean model of the elasticity matrix introduced in Equation (6)). Figure 3a presents the buckling load factor versus lateral deflection, while Figure 4c shows the deformed centerline of columns with *SLD* = 120, 140, 160, 180 and 200, respectively. The initial and deformed meshes of 120 *SLD* columns under axial compression are shown in Figure 4a,b, respectively. The critical buckling load was achieved (Table 1) and plotted in Figure 3b for comparison with Euler’s formula.

The validation of the in-house finite element tool was achieved as shown in Figure 3b. It can be observed that the finite element results are in good agreement with the Euler’s formula values. The value of the coefficient of determination R^2^ = 0.9975 was obtained. Based on this, it can be concluded that the in-house finite element tool is reliable and efficient in investigating the buckling response of the fixed-pinned end columns under axial compression. In the next section, this numerical tool is used to quantify the uncertainties related to the material properties on the buckling capacity of structural elements under compression.

### 4.2. Uncertainty Quantification

In this section, the impact of material uncertainty on the buckling capacity of columns is quantified. Three levels of fluctuations (i.e., 0.3, 0.5 and 0.7) in the elasticity matrix of the material constituting the 120 *SLD* columns were investigated. The probabilistic model for the material uncertainties is first presented using the probabilistic model, presented in Section 2.3. The validated in-house finite element tool presented in the previous section was used to quantify the statistical fluctuations of the responses in finite displacement using the Monte Carlo approach. Convergence analysis was also carried out in order to study the convergence of Monte Carlo simulations and estimate the confidence interval for the column buckling responses in finite displacement.

#### 4.2.1. Parameters of the Probabilistic Model for Material Uncertainties in Finite Displacement

**Figure 5:**
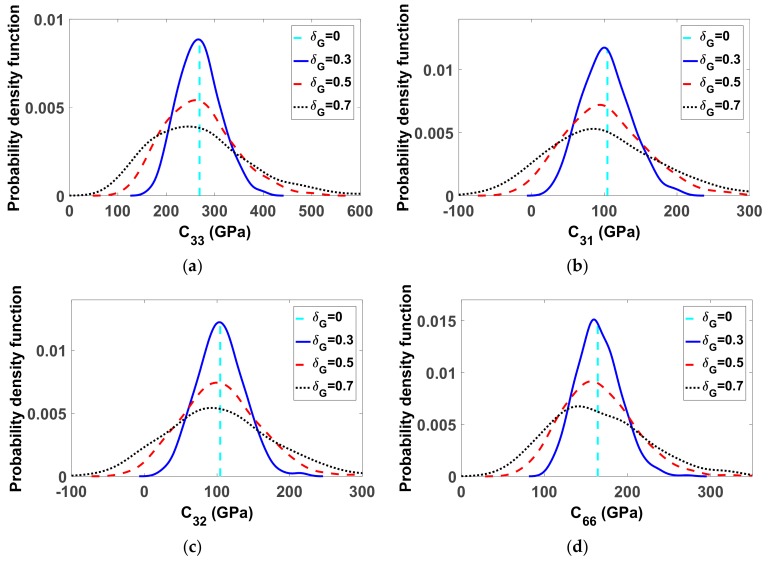
Probability density functions of several components of elasticity matrix over 500 random realizations for (**a**) C_33_, (**b**) C_31_, (**c**) C_32_ and (**d**) C_66_ (in GPa) associated to three levels of fluctuations respectively. Value of the corresponding mean model is also indicated.

The probability density functions of random entries C_ij_ of random matrix [C] were estimated using the kernel smooth density method with 500 statistical independent realizations of [C]. The mean values of entries C_ij_, given by Equation (6), are also reported in Figure 5. In Equation (33), a random realization of elasticity matrix [C] (in GPa) associated with a level of fluctuations δ*_G_* = 0.5 is presented. The eigenvalues of the elasticity matrix for this realization are all positive and are equal to 580.23, 42.61, 215.21, 199.33, 158.40 and 108.37.

(33) $$\left[C_{realization-N{}^{\circ}i}^{\delta_{G}=0.5}\right]=\left[\begin{array}{cccccc} 285.79 & 52.91 & 128.04 & 20.00 & -12.42 & -46.94\\ & 182.66 & 130.80 & 36.63 & 7.05 & -42.71\\ & & 396.40 & 58.42 & -31.24 & -74.46\\ & & & 94.48 & 17.23 & -78.42\\ & \mathrm{Sym}. & & & 142.00 & 28.71\\ & & & & & 202.83 \end{array}\right]$$

#### 4.2.2. Uncertainty Quantification

**Figure 6:**
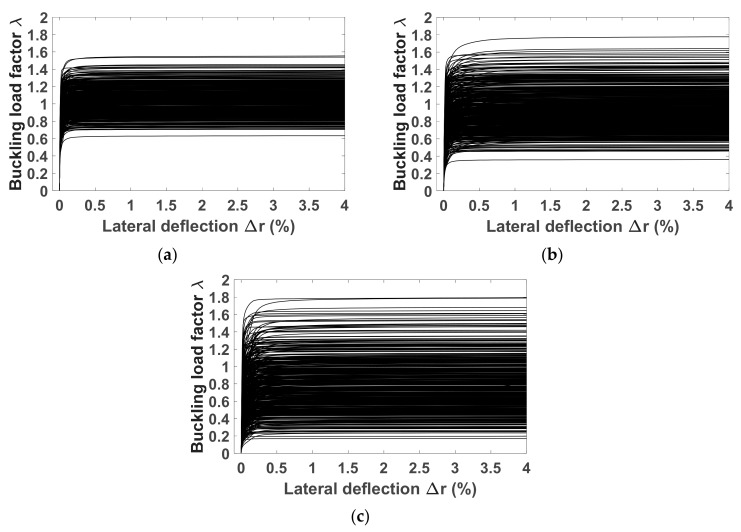
Buckling responses of columns under axial compression accounting fluctuations in the elasticity matrix for (**a**) δ*_G_* = 0.3, (**b**) δ*_G_ =* 0.5 and (**c**) δ*_G_ =* 0.7. 500 Monte Carlo runs were performed for each case.

The Monte Carlo numerical method was carried out to quantify the propagation of uncertainties on the responses of the nonlinear mechanical system. A number of N_MC_ = 1500 random statistical independent realizations of the elasticity matrix [C], corresponding to 3 different values of levels of fluctuations given by δ*_G_*, were used as input for the in-house finite element tool. Figure 6a–c presents 500 curves of buckling load factor–lateral deflection associated to δ*_G_* = 0.3, 0.5 and 0.7, respectively. It can be seen that uncertainties related to the material properties greatly affect the buckling responses of column under axial loading, that is, different load–lateral deflection relationships are obtained. Consequently, depending on each random realization of the random elasticity matrix [C], the buckling points were found at different lateral deflections. In this paper, a critical lateral deflection of 0.1% was chosen in order to normalize the impact of the material uncertainty on the global buckling response of the columns. This choice is reasonable, as the initial geometry of the column was unchanged. At 0.1% of lateral deflection (Figure 6), the buckling load factor varies from 0.6 to 1.5 with δ*_G_* = 0.3, from 0.3 to 1.6 with δ*_G_* = 0.5 and from 0.1 to 1.7 with δ*_G_* = 0.7. This means that the fluctuations of material properties cause a significant impact on the buckling capacity of columns.

**Figure 7:**
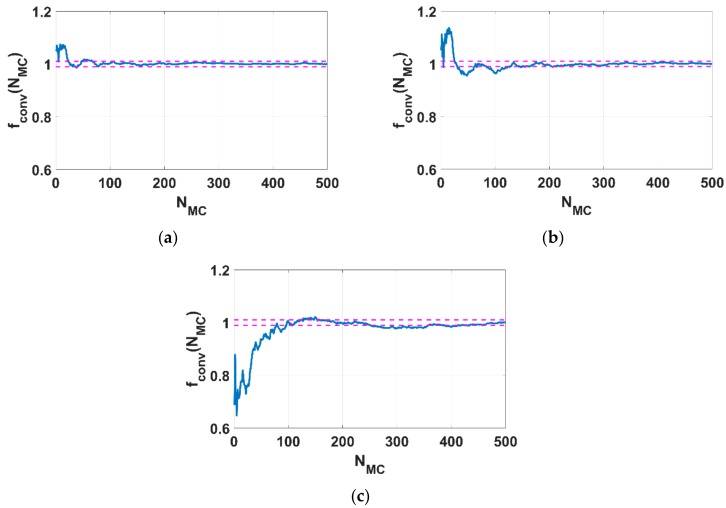
Convergence functions over 500 Monte Carlo simulations for (**a**) δ*_G_* = 0.3, (**b**) δ*_G_ =* 0.5 and (**c**) δ*_G_ =* 0.7. The discontinuous lines represent ±1% deviation around the average value.

The convergence function values of the critical buckling load factor at 0.1% of lateral deflection over 500 Monte Carlo realizations is presented in Figure 7a–c for 3 levels of fluctuations 0.3, 0.5 and 0.7, respectively. It is worth noting that this convergence function is defined in Equation (32). It is also worth noting that 500 realizations are enough to reach a convergence of the statistical estimators in average, yielding reliable results for the column buckling response. Especially in the case of δ*_G_* = 0.7, at least 400 Monte Carlo simulations were required to reach the converged statistical value in a range ±1% around the average value.

**Figure 8:**
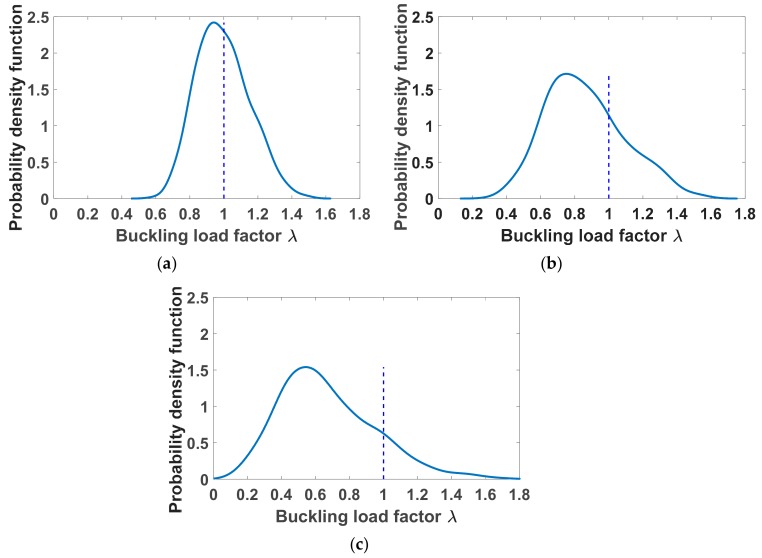
Probability density functions of critical buckling load at 0.1% of lateral deflection for (**a**) δ*_G_* = 0.3, (**b**) δ*_G_ =* 0.5 and (**c**) δ*_G_ =* 0.7.

The probability density function values of buckling load factor at 0.1% of lateral deflection are shown in Figure 8a–c associated with three levels of fluctuations δ*_G_* = 0.3, 0.5 and 0.7, respectively. The mean values, standard deviations and coefficients of variations of the random critical buckling factor *λ* are also reported in Table 2. The ratios between the coefficients of variation and the fluctuation levels were deduced and are equal to 0.5176, 0.5341 and 0.5928, respectively. Regarding the mean values, it is worth noting that increasing material uncertainty could decrease the buckling capacity of columns. The mean values of the critical buckling load are 90.43, 89.94, 77.88 and 60.51 kN when increasing the fluctuations in material uncertainty associated with four levels of 0, 0.3, 0.5 and 0.7, respectively.

**Figure 9:**
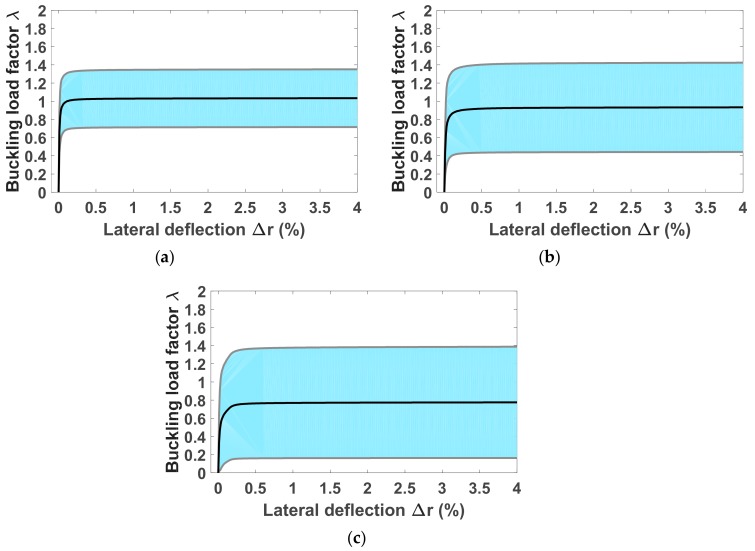
The 95% confidence intervals of column buckling behavior from 0 to 4% of lateral deflection for (**a**) δ*_G_* = 0.3, (**b**) δ*_G_ =* 0.5 and (**c**) δ*_G_ =* 0.7.

The confidence interval of 95% for the buckling behavior of columns under axial loading from 0 to 4% of lateral deflection was achieved and is shown in Figure 9a–c for the 3 levels of fluctuations, respectively. The confidence interval appeared wider when the fluctuation level (controlled by δ*_G_*) increased. Such information might be helpful in investigating the post-buckling behavior of columns.

In conclusion, the nonlinear finite element tool developed shows a good level of efficiency and robustness when combined with the Monte Carlo numerical method as a stochastic solver for quantifying the propagation of uncertainties of material properties on the buckling response of columns under axial compression. The quantification of uncertainty could be useful in accounting for the presence of fluctuations in the mechanical properties of material on the instability of columns, which is the most important failure mode of structures.

## 5. Conclusions

In this paper, a probabilistic model was introduced in order to quantify the propagation of uncertainties related to the elasticity matrices of materials constituting the structural element under compression in finite displacement. Such a probabilistic model is only parameterized by two parameters including the mean value of the elasticity matrix and a dispersion coefficient that controls the level of statistical fluctuations of the probabilistic model. An in-house finite element tool based on a Newton-Raphson solver was developed in order to track the nonlinear behavior of the columns under compressive stress. The critical Euler’s buckling load was analyzed to validate this in-house finite element tool. Results showed that the finite element analysis in finite displacement correctly computed the buckling response of the column for the mean value of the material elasticity matrix. Statistical sampling of the random elasticity matrix has been calculated in using the algebraic representation of the random elasticity matrix in the *adhoc* ensemble of random matrices. Numerical experiments were conducted for three fluctuation levels, that is, δ*_G_* = 0.3, 0.5 and 0.7. A total number of 500 Monte Carlo simulations were used to reach the convergence of the statistical estimators to quantify the propagation of material uncertainties on the buckling behavior of columns in finite displacement. Statistical analysis was performed in order to estimate the values of the mean, standard deviation, coefficient of variation and confidence intervals of critical buckling loads at these three different fluctuation levels. The results showed that the buckling behavior of a column under axial loading is greatly affected, while accounting for material uncertainties. However, the statistical convergence of Monte Carlo realizations was investigated only for the mean value. In order to better characterize the statistical behavior of variables, a convergence estimator for the standard deviation should also be addressed. In further research, methodology including the in-house finite element tool could be useful to investigate the instability of columns under compression to account not only for material uncertainties but also fluctuations in geometry of columns, loading eccentricity, residual stresses, external contact, and so on. The probabilistic model could also be extended to account for the correlation structure in heterogeneous materials using random fields of the elasticity matrix.

## Figures and Tables

**Table 1:** Summarized information to validate the developed finite element tool.

Length of Column L (m)	Radius of Circular Cross-Section R (m)	Slenderness of Column *SLD*	Critical Buckling Load Factor from FEM	Euler’s Critical Buckling Load Factor	Critical Buckling Load from FEM (kN)	Euler’s Critical Buckling Load (kN)
0.60	0.01	120	1.010	1	91.339	90.434
0.65	0.01	130	0.962	1	74.121	77.057
0.70	0.01	140	0.996	1	66.176	66.442
0.75	0.01	150	1.004	1	58.109	57.878
0.80	0.01	160	1.012	1	51.480	50.869
0.85	0.01	170	0.965	1	43.461	45.061
0.90	0.01	180	1.001	1	40.233	40.193
0.95	0.01	190	0.973	1	35.092	36.073
1.00	0.01	200	1.015	1	33.044	32.556
1.05	0.01	210	0.965	1	28.493	29.529
1.10	0.01	220	1.012	1	27.229	26.906
1.15	0.01	230	0.986	1	24.270	24.617
1.20	0.01	240	1.016	1	22.970	22.608

**Table 2:** Summary of statistical analysis of critical buckling load factor.

Level of Fluctuations in the Elasticity Matrix	Mean	Standard Deviation	Coefficient of Variation (%)
0	1	0	0
0.3	0.995	0.154	15.477
0.5	0.861	0.230	26.713
0.7	0.669	0.278	41.555
